# Elite athletes’ genetic predisposition for altered risk of complex metabolic traits

**DOI:** 10.1186/s12864-014-1199-0

**Published:** 2015-01-23

**Authors:** Lauren K Banting, Vladimir P Pushkarev, Pawel Cieszczyk, Aleksandra Zarebska, Agnieszka Maciejewska-Karlowska, M-arek Sawczuk, Agata Leońska-Duniec, Dmitry A Dyatlov, Evgeniy F Orekhov, Aleksandr V Degtyarev, Yuliya E Pushkareva, Xu Yan, Ruth Birk, Nir Eynon

**Affiliations:** Institute of Sport, Exercise and Active Living (ISEAL), Victoria University, Melbourne, Australia, VIC 8001; Ural State University of Physical Culture, Chelyabinsk, Russia; University of Szczecin, Department of Physical Culture and Health Promotion, Szczecin, Poland; Academy of Physical Education and Sport, Department of Sport Education, Gdansk, Poland; South Ural State Medical University, Chelyabinsk, Russia; Murdoch Childrens Research Institute, The Royal Children’s Hospital, Melbourne, Australia; Department of Nutrition, Faculty of Health Sciences, Ariel University, Ariel, Israel

**Keywords:** Genes, Exercise, Athletes, Obesity, Type 2 diabetes

## Abstract

**Background:**

Genetic variants may predispose humans to elevated risk of common metabolic morbidities such as obesity and Type 2 Diabetes (T2D). Some of these variants have also been shown to influence elite athletic performance and the response to exercise training. We compared the genotype distribution of five genetic Single Nucleotide Polymorphisms (SNPs) known to be associated with obesity and obesity co-morbidities (*IGF2BP2* rs4402960, *LPL* rs320, *LPL* rs328, *KCJN* rs5219, and *MTHFR* rs1801133) between athletes (all male, n = 461; endurance athletes n = 254, sprint/power athletes n = 207), and controls (all male, n = 544) in Polish and Russian samples. We also examined the association between these SNPs and the athletes’ competition level (‘elite’ and ‘national’ level). Genotypes were analysed by Single-Base Extension and Real-Time PCR. Multinomial logistic regression analyses were conducted to assess the association between genotypes and athletic status/competition level.

**Results:**

*IGF2BP2* rs4402960 and *LPL* rs320 were significantly associated with athletic status; sprint/power athletes were twice more likely to have the *IGF2BP2* rs4402960 risk (T) allele compared to endurance athletes (OR = 2.11, 95% CI = 1.03-4.30, P <0.041), and non-athletic controls were significantly less likely to have the T allele compared to sprint/power athletes (OR = 0.62, 95% CI =0.43-0.89, P <0.0009). The control group was significantly more likely to have the *LPL* rs320 risk (G) allele compared to endurance athletes (OR = 1.26, 95% CI = 1.05-1.52, P <0.013). Hence, endurance athletes were the “protected” group being significantly (p < 0.05) less likely to have the risk allele compared to sprint/power athletes (*IGF2BP2* rs4402960) and significantly (p < 0.05) less likely to have the risk allele compared to controls (*LPL* rs320). The other 3 SNPs did not show significant differences between the study groups.

**Conclusions:**

Male endurance athletes are less likely to have the metabolic risk alleles of *IGF2BP2* rs4402960 and *LPL* rs320, compared to sprint/power athletes and controls, respectively. These results suggest that some SNPs across the human genome have a dual effect and may predispose endurance athletes to reduced risk of developing metabolic morbidities, whereas sprint/power athletes might be predisposed to elevated risk.

## Background

Complex metabolic diseases such as Obesity and Type 2 Diabetes (2TD) and physical activity levels have long been recognised as being closely-related. For instance, it has been shown that elite athletes or former elite athletes tend to have longer life expectancies, and lower risks of complex metabolic diseases such as obesity and T2D, than matched sedentary controls [1-3]. Genetic factors seem to play a role in elite athlete development, on one hand [4,5], and the predisposing for complex metabolic diseases, on the other hand [6]. Recently, we [6] and others [7,8] hypothesised that genetic Single Nucleotide Polymorphisms (SNPs), including SNPs identified in Genome Wide Association Studies (GWAS) that have been associated with increased risk for complex metabolic diseases, would also be candidates to influence athletic performance/physical activity levels.

The A/T polymorphism (rs9939609) in the fat mass and obesity associated (*FTO*) gene, was discovered in two separate GWAS [9,10], and is an example of specific variant associated with obesity, 2TD, and physical activity levels. Recent meta-analysis combining data from adults and children, and an adolescent population (overall 54 studies of n = 218,166 and n = 19,268, respectively) have shown that physically active people with the *FTO* risk allele are 30% less likely to be obese compared to their inactive counterparts [11]. *Visfatin*, a recently discovered adipokine that contributes to glucose and obesity-related conditions, is another gene that potentially influences both exercise-related phenotypes and complex metabolic diseases. rs4730153 within the *Visfatin* was associated with aerobic exercise training-induced changes in glucose and obesity-related phenotypes [12]. The peroxisome proliferator-activated receptor gamma coactivator1α (*PPARGC1A*) Gly482Ser SNP was also associated with increased risk of obesity and type 2 diabetes [13] on one hand, and with elite athletic performance [14-17], on the other hand.

The outcomes of the abovementioned studies assist with understanding the genomic link between complex metabolic diseases and athletic performance; however the widely accepted hypothesis is that there are likely to be many other uncovered variants with dual effects. In that sense, elite athletes represent the end point of the human physical activity continuum with a “rare” and distinguished phenotype, and hence are an excellent model to study.

Potential obesity and T2D-related genetic variants that may influence athletic performance as well are located in the *IGF2BP2*, *LPL*, *KCJN*, and *MTHFR* genes. *IGF2BP2* rs4402960 G > T variant is associated with predisposition to T2D and obesity. GWAS studies have indicated that the risk allele for T2D and obesity is the T allele. Animal model and human studies implicate this variant with reduced beta-cell function, insulin secretion and sensitivity and with raised fasting glucose levels [18-20]. Importantly, recent studies suggest a potential role for IGF2BP2 in skeletal muscle cell proliferation and differentiation [21]. *LPL* rs320 and rs328 SNPs have been associated with plasma lipids levels, through the protein’s role in the uptake of Free Fatty Acids (FFA) from the plasma to tissues, including muscle cells [22-24]. Thus, it has been hypothesised that these SNPs may alter the availability of FFA to muscle cells and to the utilization of fat by muscles. The obesity risk allele/genotype for both rs320 and rs328 are G allele and the GG genotype. KCNJ11 is an ATP-sensitive K+ (KATP) channel, which couples cell metabolism with membrane excitability in various cell types, including muscle cells. The protein’s known function is mainly related to diabetes phenotypes [25]. However, it was also found to be association with impaired exercise stress response in several models. The E23K SNP at codon 23 of the *KCNJ11* gene (rs5219) results in substitution of glutamic acid to lysine, and may cause modest reductions in ATP sensitivity, which could influence muscle response to exercise. The metabolic risk allele/genotype in rs5219 is T/TT. MTHFR is a key enzyme in one carbon cycle. *MTHFR* C677T SNP results in elevated plasma homocysteine, which has been linked to reduced mobility and muscle functioning in the elderly (women) and has been associated with T2D. The risk allele/genotype in rs1801133 is T/TT [26,27].

Therefore, we studied the association between these five genetic variants associated with both obesity and obesity co-morbidities (*IGF2BP2* rs4402960, *LPL* rs320, *LPL* rs328, *KCJN* rs5219, and *MTHFR* rs1801133) and elite athletic status in a relatively-large cohort (n = 929, from Poland and Russia) of sprint/power and endurance athletes. We also examined the association between these variants and athletic status according to the athletes’ level of competition (‘elite’ and ‘national’ level). We hypothesised that the obesity and/or co-morbidities risk allele/genotype in each of these variants would be underrepresented in elite athletes compared to controls.

## Methods

The study was approved by the Pomeranian Medical University Ethics Committee, Poland, and the Ural State University of Physical Culture, Russia, and written informed consent was obtained from each participant. The study complied with the guidelines set out in the Declaration of Helsinki and the ethics policy of the Szczecin University [28].

### Participants

A total of 929 male participants from Russia (n = 281) and Poland (n = 648) were involved in the study. The Russian participants included 177 athletes (mean age = 26.3, SD = 10.3) and 104 unrelated sedentary controls (mean age = 31.2, SD = 10.4). The Polish participants were 208 athletes (mean age = 28.6, SD = 6.2) and 440 unrelated sedentary controls (mean age = 22.4, SD = 2.5). All athletes were ranked in the top 10 nationally in their sport discipline and grouped as being either ‘elite-level’ or ‘national-level’ based on their best personal performance. Those in the elite group had participated in international competitions such as World and European Championships, and/or Olympic Games, whereas those in the national-level group had participated in national competitions only. Athletes were further classified as endurance (events requiring predominantly aerobic energy production including long distance and duration events or sprint/power athletes (events requiring predominantly anaerobic energy production).

#### Russian athletes

This group included 70 (elite n = 10; 14%) endurance athletes and 107 (elite n = 44; 41%) sprint/power athletes. Athletes in classified as endurance athletes included cross country skiers (n = 38), marathon runners (n = 2), rowers (n = 13), 1500-5000 m runners (n = 4), 5000/10 000 m long distance skaters (n = 6), 3000 m steeple chase (n = 1), 1500 m swimmers (n = 2) and walkers (n = 4). Athletes classified as sprint/power athletes came from sports including ski-cross/ alpine skiing (n = 2), discus throw (n = 1), Greco-roman wrestling (n = 10), pole vault (n = 1), 200 m sprint (n = 2), 100 m sprint (n = 10), 400 m sprint (n = 1), 500 m short distance skating (n = 23), 50/100 m short distance swimming (n = 8), and power lifters (n = 49).

#### Polish athletes

This group included 108 (elite n = 65; 60%) endurance athletes and 100 (elite n = 64; 64%) sprint/power athletes. Athletes classified as endurance athletes included canoeists (n = 10), cross country skiers (n = 2), cyclists (n = 14), 10 00 m/marathon runners (n = 25), rowers (n = 41), 1500 m swimmers (n = 10), and triathletes (n = 6). The sprint/power athletes included 100/200 m runners (n = 34), archers (n = 4), weight/ power lifters (n = 42), high jumpers (n = 1), javelin throwers (n = 1), long jumpers (n = 4), vaulters (n = 3), shooters (n = 1), shot putters (n = 5), 500 m short-distance skaters (n = 1) sky jumpers (n = 2) and 50/100 m swimmers (n = 2).

### Genotyping

In the Polish cohort, Genomic DNA was isolated from buccal epithelium using GenElute Mammalian Genomic DNA Miniprep Kit (Sigma, Hamburg, Germany) according to the manufacturer’s instructions. In the Russian cohort, Genomic DNA was isolated from buccal epithelium or peripheral blood, during the years 2011-2013, using the Diatom™ DNA Prep kit (Cat. # D 1025, IsoGene Lab Ltd, Russia). Genotyping was performed as previously described [29]. In the Russian cohort, genotyping of four SNPs (*IGFBP2* rs4402960, *KCJN11* rs5219, *LPL* rs320 and rs328) was performed by Single-Base Extension (SBE) method. The sequence surrounding each SNP was obtained from the Genome Reference Consortium Human genome build 37 assembly from the Ensembl Project (www.ensembl.org). The Primer3web software v. 4.0.0 (http://bioinfo.ut.ee/primer3) was used for designing the PCR primers. Genotyping of the *MTHFR* rs1801133 polymorphism was performed by using a TaqMan® SNP Genotyping Assay with a StepOne™ Real-Time PCR System (Applied Biosystems, Foster city, CA, USA). The assay ID was C___1202883_20. The results were analysed by using TaqMan® Genotyper Software (Applied Biosystem). K562 DNA High Molecular Weight from Promega Corp. (Cat # DD2011, Madison, WI, USA) served as a positive control sample. Genetic profile of the K562 DNA was as follow: *IGFBP2* rs4402960 – G/G, *KCJN11* rs5219 – C/T, *LPL* rs320 – G/G, *LPL* rs328 – G/C, and *MTHFR* rs1801133 – G/G.

In the Polish cohort, all samples were genotyped in duplicate using allelic discrimination assays with Taqman® probes (Applied Biosystems, Carlsbad, California, USA) on a CFX96 Touch™ Real-Time PCR Detection System (Bio-Rad, Hercules, California, USA). To discriminate rs4402960, rs320, rs328, rs5219, and rs1801133 alleles, TaqMan® Pre-Designed SNP Genotyping Assays were used (assay IDs: C___2165199_10, C___1843003_10, C____901792_1_, C__11654065_10, C___1202883_20 respectively), including appropriate primers and fluorescently labeled (FAM and VIC) MGB™ probes to detect the alleles.

### Genotyping reliability across two laboratories

As previously described [29] genotyping was performed in duplicate in the same Laboratory for accuracy. Two independent investigators have called the genotyping score in each laboratory-100% of the genotypes could be called. For the purpose of results reliability across two laboratories in two different countries (Russia and Poland), different DNA samples (one for each SNP, positive or negative controls) were shipped from Russia to Poland and were genotyped by TaqMan assays. The results of the genotyping were in 100% agreement across the two laboratories.

### Statistical analysis

Chi squared tests were used to test for the presence of Hardy-Weinberg equilibrium (HWE). HWE was tested separately for each SNP. Genotype frequencies were compared according to athletic status (i.e. controls, endurance, or sprint/power athlete) using Fisher’s exact test. Multinomial logistic regression analyses were conducted to assess the association between genotype and athletic status/competition level. Nationality was adjusted for in the first stage of analysis as there were nationality distribution differences in each athletic status groups and the control group. The homozygous non-risk allele genotype was chosen as the reference genotype for each analysis, with comparisons made to the heterozygous genotype and the homozygous risk allele genotype (co-dominant models). Additional comparisons were made to assess the dominant and recessive models, as described in our work [30]. Significance between these planned comparisons was accepted when p ≤ 0.05. Odds ratios with 95% confidence intervals were also calculated for estimation of the risk effect.

## Results

Genotype frequencies distribution for *IGFBP2* rs4402960, *LPL* rs320, *LPL* rs328, *KCJN* rs5219, and *MTHFR* rs1801133 amongst all participants is presented in Tables 1, 2, 3, 4, and 5. In the pooled cohort of Russian and Polish controls, genotype distributions for each of the five SNPs was in agreement with HWE (p-value > 0.05). In the Polish cohort *LPL* rs320 deviated from HWE (P = 0.026), however LPL rs320 was in agreement with HWE in the Russian cohort (p = 0.7) (Table 2). The analyses for all the SNPs was performed on the pooled cohort, hence the HWE deviation in the Polish cohort had no effect on the results.

**Table 1:** **Genotype frequency distributions for** ***IGFBP2*** **rs4402960**

	**Polish (n = 648)**			**Russian (n = 281)**		
	**Endurance**	**Sprint/Power**	**Control**	**Endurance**	**Sprint/Power**	**Control**
All (N)	108	100	440	70	107	104
TT	10 (9%)	16 (16%)	42 (10%)	4 (6%)	13(12%)	11 (11%)
GT	47 (44%)	40 (40%)	163 (37%)	37 (53%)	58 (54%)	39 (38%)
GG	51 (47%)	44 (44%)	235 (53%)	29 (41%)	36 (34%)	54 (52%)
MAF	0.31	0.36	0.28	0.32	0.39	0.29
HWE-P value	0.987	0.419	0.223	0.208	0.368	0.623
Elite (N)	65	64	-	10	44	-
TT	4 (6%)	14 (22%)		2 (20%)	7 (16%)	
GT	28 (43%)	20 (31%)		5 (50%)	22 (50%)	
GG	33 (51%)	30 (47%)		3 (30%)	15 (34%)	
MAF	0.28	0.38		0.45	0.41	
National Level (N)	43	36		60	63	-
TT	6 (14%)	2 (6%)		2 (3%)	6 (10%)	
GT	19 (44%)	20 (56%)		32 (53%)	36 (57%)	
GG	18 (42%)	14 (39%)		26 (43%)	21 (33%)	
MAF	0.36	0.33		0.30	0.38	

*Note:* TT is identified as the risk genotype.

**Table 2:** **Genotype frequency distributions for** ***LPL*** **rs320**

	**Polish (n = 648)**			**Russian (n = 281)**		
	**Endurance**	**Sprint/Power**	**Control**	**Endurance**	**Sprint/Power**	**Control**
All (N)	108	100	440	70	107	104
GG	8 (7%)	5 (5%)	21 (5%)	4 (6%)	13 (12%)	8 (8%)
GT	35 (32%)	46 (46%)	196 (45%)	26 (37%)	37 (35%)	48 (46%)
TT	65 (60%)	49 (49%)	223 (51%)	40 (57%)	57 (53%)	48 (46%)
MAF	0.24	0.28	0.27	0.24	0.29	0.31
HWE-P value	0.573	0.370	0.026	0.996	0.222	0.697
Elite (N)	65	64	-	10	44	-
GG	3 (5%)	4 (6%)		0	6 (14%)	
GT	22 (33%)	30 (47%)		6 (60%)	12 (27%)	
TT	40 (62%)	30 (47%)		4 (40%)	26 (59%)	
MAF	0.22	0.30		0.30	0.27	
National Level (N)	43	36		60	63	-
GG	5 (12%)	1 (3%)		4 (7%)	6 (10%)	
GT	13 (30%)	16 (44%)		20 (33%)	29 (46%)	
TT	25 (58%)	19 (53%)		36 (60%)	28 (44%)	
MAF	0.27	0.25		0.23	0.33	

*Note:* GG is identified as the risk genotype.

**Table 3:** **Genotype distributions for** ***LPL*** **rs328**

	**Polish (n = 648)**			**Russian (n = 281)**		
	**Endurance**	**Sprint/Power**	**Control**	**Endurance**	**Sprint/Power**	**Control**
All (N)	108	100	440	70	107	104
GG	1(1%)	1 (1%)	6 (1%)	0	0	0
GC	14 (13%)	17 (17%)	75 (17%)	8 (11%)	13 (12%)	16 (15%)
CC	93 (85%)	82 (82%)	359 (82%)	62 (89%)	94 (88%)	88 (85%)
MAF	0.07	0.10	0.10	0.94	0.94	0.92
HWE-P value	0.849	0.994	0.661	0.879	0.799	0.697
Elite (N)	65	64	-	10	44	-
GG	0	0		0	0	
GC	7 (11%)	10 (16%)		1 (10%)	10 (23%)	
CC	58 (89%)	54 (84%)		9 (90%)	34 (77%)	
MAF	0.05	0.08		0.95	0.88	
National Level (N)	43	36		60	63	-
GG	1 (2%)	1 (1%)		0	0	
GC	7 (16%)	7 (19%)		7 (23%)	3 (5%)	
CC	35 (81%)	28 (80%)		53 (77%)	60 (95%)	
MAF	0.10	0.13		0.94	0.98	

*Note:* GG is identified as the risk genotype.

**Table 4:** **Genotype frequency distributions for** ***KCJN*** **rs5219**

	**Polish (n = 648)**			**Russian (n = 281)**		
	**Endurance**	**Sprint/Power**	**Control**	**Endurance**	**Sprint/Power**	**Control**
All (N)	108	100	440	70	107	104
TT	14 (13%)	12 (12%)	60 (14%)	11 (16%)	13 (12%)	13 (13%)
CT	44 (41%)	50 (50%)	178 (40%)	26 (37%)	46 (42%)	49 (47%)
CC	50 (46%)	38 (38%)	202 (46%)	33 (47%)	49 (46%)	42 (40%)
MAF	0.33	0.37	0.33	0.34	0.336	0.36
HWE-P value	0.687	0.769	0.127	0.339	0.907	0.976
Elite (N)	65	64	-	10	44	-
TT	7 (11%)	6 (9%)		2 (20%)	7 (16%)	
CT	25 (37%)	32 (50%)		3 (30%)	17 (39%)	
CC	33 (51%)	26 (41%)		5 (50%)	20 (45%)	
MAF	0.30	0.34		0.35	0.35	
National Level (N)	43	36		60	63	-
TT	7 (16%)	6 (17%)		9 (15%)	6 (10%)	
CT	19 (44%)	18 (50%)		23 (38%)	29 (46%)	
CC	17 (40%)	12 (33%)		28 (47%)	28 (44%)	
MAF	0.38	0.42		0.34	0.33	

*Note:* TT is identified as the risk genotype.

**Table 5:** **Genotype frequency distributions for** ***MTHFR*** **rs1801133**

	**Polish (n = 648)**			**Russian (n = 281)**		
	**Endurance**	**Sprint/Power**	**Control**	**Endurance**	**Sprint/Power**	**Control**
All (N)	108	100	440	70	107	104
TT	12 (11%)	9 (9%)	33 (8%)	4 (6%)	11 (10%)	9 (9%)
CT	43 (40%)	40 (40%)	186 (42%)	30 (43%)	52 (49%)	38 (37%)
CC	53 (49%)	51 (51%)	221 (50%)	36 (51%)	44 (41%)	57 (55%)
MAF	0.31	0.29	0.29	0.27	0.35	0.27
HWE-P value	0.770	0.960	0.772	0.78	0.745	0.767
Elite (N)	65	64	-	10	44	-
TT	11 (17%)	9 (14%)		2 (20%)	4 (9%)	
CT	24 (37%)	25 (39%)		5 (50%)	25 (57%)	
CC	30 (46%)	30 (47%)		3 (30%)	15 (34%)	
MAF	0.35	0.34		0.45	0.38	
National Level (N)	43	36	-	60	63	-
TT	1 (2%)	0		2 (3%)	7 (11%)	
CT	19 (44%)	15 (42%)		25 (42%)	27 (43%)	
CC	23 (53%)	21 (58%)		33 (55%)	29 (46%)	
MAF	0.24	0.21		0.24	0.33	

*Note:* TT is identified as the risk genotype.

*IGF2BP2* rs4402960 was significantly associated with athletic status (Table 6). The control participants were less likely than sprint/power athletes to have the TT (increased risk) genotype compared to GG genotype (OR: 0.62 [0.43-0.89]; p = 0.009), and TT and GT combined (OR: 0.59 [0.42- 0.84]; p = 0.003). The sprint/power athletes were more likely than endurance to be TT compared to GG (OR: 2.11 [1.01-3.95]; p = 0.041), and GT and TT combined (OR: 2.00 [1.01- 3.95]; p = 0.045).

**Table 6:** **Ratios of genotype distributions according to athlete type for** ***IGFBP2*** **rs4402960**

**Sport type**	**GG (ref)**	**GT**			**TT**			**TT (GT&GG ref)**			**TT & GT (GG ref)**		
	**OR**	**OR**	**CI**	***p***	**OR**	**CI**	***p***	**OR**	**CI**	***p***	**OR**	**CI**	***p***
Control vs. Sprint/Power	1	0.62	0.43-0.89	0.009	0.51	0.30-0.88	0.016	0.65	0.39-1.08	0.094	0.59	0.42-0.84	0.003
Sprint/Power vs. Endurance	1	1.10	0.71-1.70	0.661	2.11	1.03-4.30	0.041	2.00	1.01-3.95	0.045	1.24	0.83-1.88	0.298
Control vs. Endurance	1	0.83	0.69-0.99	0.041	1.02	0.74-1.41	0.887	1.11	0.82-1.53	0.487	0.86	0.72-1.02	0.086

*Note:* OR: Odds Ratio; CI: Confidence intervals; *p*: 2 tailed *p* value. Significance is assumed when *p* < 0.05.

IGFBP2 rs4402960.

*LPL* rs320 was also significantly associated with athletic status. Table 7 shows that the control group is more likely than the endurance athletes to have the GT genotype compared to TT genotype (OR: 1.26 [1.05-1.52]; p = 0.013). Controls are also more likely than endurance to have the risk-related GG&GT genotypes compared to the TT genotype (OR: 1.22 [1.03-1.46]; p = 0.024).

**Table 7:** **Ratios of genotype distributions according to athlete type for** ***LPL*** **rs320**

**Sport type**	**TT (ref)**	**GT**			**GG**			**GG (GT& TT ref)**			**GG & GT (TT ref)**		
	**OR**	**OR**	**CI**	***p***	**OR**	**CI**	***p***	**OR**	**CI**	***p***	**OR**	**CI**	***p***
Control vs. Sprint/Power	1	1.13	0.79-1.61	0.499	0.79	0.40-1.55	0.494	0.75	0.39-1.44	0.384	1.07	0.77-1.51	0.681
Sprint/Power vs. Endurance	1	1.37	0.89-2.11	0.152	1.42	0.65-3.11	0.386	1.25	0.58-2.69	0.570	1.38	0.92-2.07	0.124
Control vs. Endurance	1	1.26	1.05-1.52	0.013	1.00	0.69-1.44	0.996	0.91	0.63-1.29	0.510	1.22	1.03-1.46	0.024

*Note:* OR: Odds Ratio; CI: Confidence intervals; *p*: 2 tailed *p* value. Significance is assumed when *p* < 0.05.

There were no differences between the studied groups and the control group across *LPL* rs328, *KCJN* rs5219, and *MTHFR* rs1801133 genotypes (Tables 8, 9 and 10). Furthermore, no significantly greater/lesser odds ratios were observed for any of the genotypes in either competition level.

**Table 8:** **Ratios of genotype distributions according to athlete type for** ***LPL*** **rs328**

**Sport type**	**CC (ref)**	**GC**			**GG**			**GG (GC&CC ref)**			**GG & GC (CC ref)**		
	**OR**	**OR**	**CI**	***p***	**OR**	**CI**	***p***	**OR**	**CI**	***p***	**OR**	**CI**	***p***
Control vs. Sprint/Power	1	1.22	0.59-2.52	0.594	1.17	0.49-2.80	0.728	0.97	0.55-1.71	0.924	1.21	0.59-2.49	0.602
Sprint/Power vs. Endurance	1	1.09	0.45-2.65	0.858	0.79	0.26-2.44	0.686	0.74	0.35-1.55	0.419	1.06	0.44-2.56	0.904
Control vs. Endurance	1	1.14	0.76-1.72	0.518	0.96	0.59-1.55	0.957	0.85	0.63-1.14	0.270	1.11	0.75-1.65	0.605

*Note:* OR: Odds Ratio; CI: Confidence intervals; *p*: 2 tailed *p* value. Significance is assumed when *p* < 0.05.

**Table 9:** **Ratios of genotype distributions according to athlete type for** ***KCJN*** **rs5219**

**Sport type**	**CC (ref)**	**TC**			**TT**			**TT (TC&CC ref)**			**TT & TC (CC ref)**		
	**OR**	**OR**	**CI**	***p***	**OR**	**CI**	***p***	**OR**	**CI**	***p***	**OR**	**CI**	***p***
Control vs. Sprint/Power	1	0.85	0.59-1.22	0.371	1.02	0.59-1.76	0.947	1.11	0.66-1.85	0.696	0.88	0.63-1.36	0.477
Sprint/Power vs. Endurance	1	1.36	0.88-2.11	0.165	0.96	0.51-1.82	0.911	1.26	0.84-1.89	0.274	0.83	0.46-1.51	0.538
Control vs. Endurance	1	1.07	0.89-1.29	0.498	1.01	0.77-1.31	0.968	0.96	0.76-1.25	0.845	1.05	0.88-1.25	0.575

*Note:* OR: Odds Ratio; CI: Confidence intervals; *p*: 2 tailed *p* value. Significance is assumed when *p* < 0.05.

**Table 10:** **Ratios of genotype distributions according to athlete type for** ***MTHFR*** **rs1801133**

**Sport type**	**AA (ref)**	**GA**			**GG**			**GG (GA& AA ref)**			**GG & GA (AA ref)**		
	**OR**	**OR**	**CI**	***p***	**OR**	**CI**	***p***	**OR**	**CI**	***p***	**OR**	**CI**	***p***
Control vs. Sprint/Power	1	1.11	0.59-2.06	0.753	1.32	0.71-2.45	0.374	1.22	0.87-1.71	0.256	1.22	0.67-2.20	0.519
Sprint/Power vs. Endurance	1	0.97	0.47-2.01	0.932	0.84	0.41-1.74	0.647	0.87	0.58-1.30	0.488	0.90	0.45-1.81	0.768
Control vs. Endurance	1	1.09	0.79-1.50	0.610	1.11	0.81-1.53	0.508	1.10	0.81-1.50	0.536	1.04	0.87-1.23	0.670

*Note:* OR: Odds Ratio; CI: Confidence intervals; *p*: 2 tailed *p* value. Significance is assumed when *p* < 0.05.

Finally, Tables 1, 2, 3, 4, and 5 show the percentage of genotypes present in elite-level and national-level athletes according to nationality and athletic status. No significant genotype differences were observed between elite-level and national-level athletes in all SNP and across nationalities (all *p* > 0.05).

## Discussion

We studied the association between five obesity and co-morbidities-related genetic variants (*IGF2BP2* rs4402960, *LPL* rs320, *LPL* rs328, *KCJN* rs5219, and *MTHFR* rs1801133) and athletic status in a well-defined (athletic level, ethnicity, gender) athletic population. We found a significant association between *IGF2BP2* rs4402960 and *LPL* rs320 and athletic status; endurance athletes are less likely to have the metabolic risk *IGF2BP2* T and *LPL* rs320 G alleles compared with sprint/power athletes and controls, respectively*.* These results suggest that male endurance athletes might be genetically predisposed toward a reduced risk of developing metabolic morbidities, compared with sprint/power athletes and the general population.

Previous studies have demonstrated that genetic variants associated with predisposition to obesity are also associated with responsiveness to exercise training [31-36]. Only a handful of variants, however, were replicated in multiple cohorts mainly due to variability in exercise training level, different ethnicity, gender, age, and cohorts with different metabolic states. To overcome some of the past studies challenges, including variability in physical activity status, different ethnicity and gender, we recruited a relatively- large cohort of Caucasians athletes with a well-defined athletic phenotype.

IGF2BP2*,* also referred to as IMP2, belongs to a mRNA-binding protein family involved in the development and stimulation of insulin action. The IGF binding protein family plays a role in modulation of IGF2 translation in a tissue-specific and developmental manner [37,38]. Several GWAS have found that carriers of the minor alleles in SNPs rs1470579 and rs4402960 have moderately increased risk for T2D. This association was confirmed across different ethnicities and populations [37-46]. Furthermore, a recent meta-analysis of 48 independent studies confirmed this association in European, East Asian and South Asian populations [47].

The intron 2 G > T substitution in the *IGF2BP2* rs4402960 is particularly interesting and has attracted the most attention in obesity and T2D studies. The SNP is located in the second, large *IGF2BP2* intron; thus, it is not yet clear how it generates its effect, whether directly through regulatory effects or indirectly through other genes. However, in the context of T2D, animal model and human studies implicate a role for this variant in beta-cell function, insulin secretion and sensitivity, and with elevated fasting glucose levels [18-20]. Importantly, recent studies suggest a potential role for IGF2BP2 protein in skeletal muscle cell proliferation and differentiation [21]. In the present study we have demonstrated that endurance athletes are less likely to have the metabolic risk alleles of *IGF2BP2* compared to sprint/power athletes who are twice as much likely to have the metabolic risk allele (homozygote) compared to endurance athletes.

An additional finding in the present study is that endurance athletes are less likely to have the metabolic risk, G allele, of *LPL* rs320, compared with controls. LPL plays a pivotal role in lipid metabolism by hydrolysing triglyceride -rich lipoproteins. Dysfunction of LPL protein increased the susceptibility for developing several common diseases, including atherosclerosis and obesity [22,47-50]. *LPL* rs320 or *Hind*III (intron 8) is a common variant in the *LPL* gene that has been associated with plasma lipid profile [22,24,51-54]. Although a large number of variants have been identified in the *LPL* gene, rs320 is of particular interest because of its common occurrence in many populations. Due to *LPL* rs320′s location within an intron, it was not initially considered functional but rather in linkage disequilibrium with a putative functional variant, such as *LPL* rs328. However, recent findings suggests that the *LPL* rs320 may be functional by altering the binding of a transcription factor and impacting *LPL* expression [49]. We found that sedentary controls are more likely to have the risk variant compared with endurance athletes and thus, might in more risk to develop elevated blood lipids and Cardio Vascular Disease [55].

A possible explanation to the underrepresentation of metabolic diseases risk alleles in endurance athletes arising from studies that evaluated the overall risk of athletes for metabolic and cardiovascular disease. Guo et al., [56] have shown that professional strength-oriented athletes at the heaviest-weight-class are at a significant increased risk for cardiometabolic disease compared with those at all other weight categories. Similarly, Urho et al., [57] found that, compared with controls, strength/power-sports athletes had a higher risk for high body mass index (BMI), whereas former endurance athletes had the lowest odds ratios for T2D and ischemic heart disease. These studies reinforce our hypothesis that endurance athletes would be at lower risk for complex metabolic diseases compared to sprint/power athletes, and controls, and genetics might be, at least partly, behind these differences.

## Conclusions

In conclusion, we found a significant association between *IGF2BP2* and *LPL* SNPs and athletic status in males: endurance athletes are less likely to have the metabolic risk alleles of *IGF2BP2* rs4402960 and *LPL* rs320, compared to sprint/power athletes and controls*.* These results suggest that some SNPs across the human genome have dual effect and may predispose endurance athletes to reduced risk of developing metabolic morbidities, whereas sprint/power athletes might be predisposed to elevated risk. These results need to be confirmed in athlete cohorts with different geographical backgrounds. Future studies should also measure obesity-related intermediate phenotypes, such as fasting blood glucose levels and plasma lipids that could lend support for the associations.
